# New approaches to the study of immune responses in humans

**DOI:** 10.1007/s00439-020-02129-3

**Published:** 2020-02-10

**Authors:** Petter Brodin

**Affiliations:** 1 Science for Life Laboratory, Department of Women’s and Children’s Health, Karolinska Institutet, 17121 Solna, Sweden; 2 Unit of Pediatric Rheumatology, Karolinska University Hospital, 17176 Solna, Sweden

## Abstract

The human immune system consists of multiple, layered mechanisms of sensing and responding to cellular stress, infection and tissue damage to ensure defense from pathogens, maintenance of tissue homeostasis, and the integrity of the holobiont. Every single cell in the body has a role to play, but a few dozen, specialized white blood cells are particularly important in this respect. Understanding the overall state of this multifaceted system in a single individual is challenging, and we are only beginning to do this across populations of individuals, to understand the vast range of inter-individual variation, and the influences of genes and environmental factors that collectively shape the immune system in a given individual. We are also only beginning to understand the changes occurring within this system over time, and how this relates to health and disease susceptibility. Several technological breakthroughs in recent years have enabled these developments and the emergence of a new, complementary approach to studying human immune systems, namely systems immunology. In this paradigm, the focus is shifted from the understanding of individual immune system components and their mechanisms of action, towards analyses of cell–cell interactions, and mechanisms of coordination and regulation within the human immune system.

## The human immune system in health and disease

The immune system plays a role in every human condition, whether it involves wound healing, fending off invading pathogens, rejecting damaged and malignant cells, ensuring successful reproduction or responding to internal stressors (Hayday and Peakman 2008; Davis et al. 2017). Across disciplines of clinical medicine, traditionally, devoid of immunological jargon, clinicians and researchers are now increasingly discussing the importance of inflammation, autoimmune reactions, symbiotic immune–microbe relationships, and the possibilities of modulating immune system components to relieve disease. Notable examples include the large-scale effort to prevent secondary myocardial infarction in patients at high risk, by blocking the pro-inflammatory mediator IL-1b with a monoclonal antibody within the CANTOS trial showing promising result (Ridker et al. 2017). Similarly, a growing number of researchers are betting on the immune system for understanding and treating neuro-degenerative diseases such as Alzheimer’s and Parkinson’s disease (Hammond et al. 2019). Finally, the virtual explosion in investment, activity and popular interest within the field of cancer immunotherapy is an illustrative example of this growing interest in modulating and harnessing of the power of the human immune system in recent years (Khalil et al. 2016). At the same time, all are not trouble free in the world of human immunology. The most important immunological achievement in history, namely the prevention of millions of deaths by virtue of vaccination, has come into question by a growing number of individuals refusing vaccines leading to the resurgence of diseases such as measles. A better understanding of the mechanisms of successful vaccination will be important to improve vaccine efficacy, and developing simpler vaccine regimens, with fewer booster vaccinations, and this will be important to further counteract the anti-vaccine movements drumming up unfounded claims of vaccine adverse effects. Common to all these applications of human immunology is that better methods for describing the composition and function of human immune systems will be important for further advancements and such methods are the focus of the current review.

## Technical advances and systems immunology

**Fig. 1:**
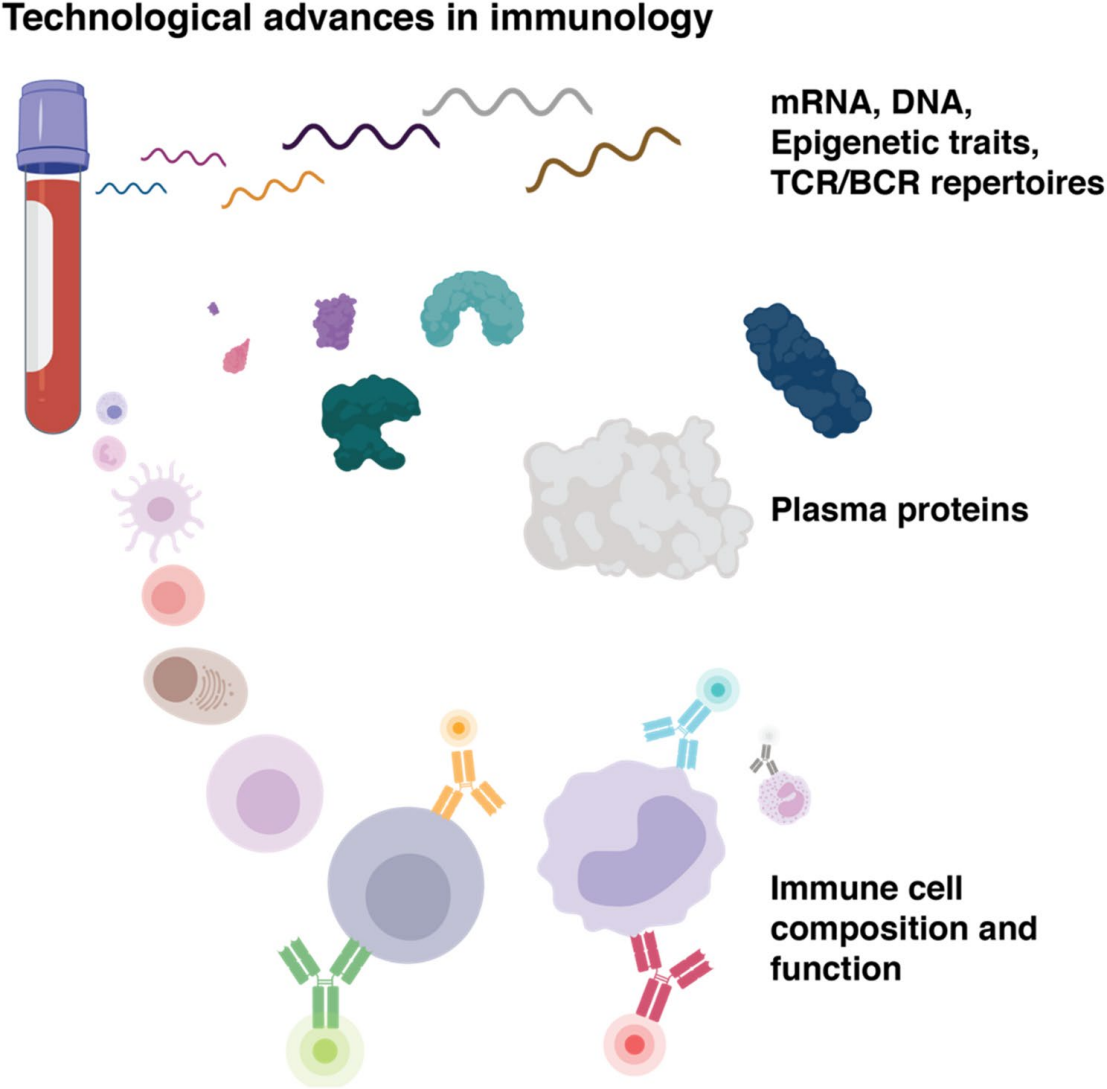
Technological advances in human immunology. Sequencing-based methods are rapidly evolving and now enable single-cell analyses of mRNA, DNA, epigenetics and variable lymphocyte receptor sequences and clonality. High-dimensional plasma protein analyses allow for hundreds of proteins to be quantified in a sample, and cytometric advances enable characterization and sorting of all possible subpopulations of immune cells

Systems immunology is a buzz word denoting the application of theories and technologies from the field of systems biology towards the study of immunological reactions. The rationale behind this approach is that the immune system is a distributed system, without any master regulator, and its responses arise as a consequence of coordinated activities of many different, specialized immune cell populations. To understand such responses, and be able to predict their outcome, these different cell populations and proteins, must be measured simultaneously in the same sample (Davis and Brodin 2018). This is now possible thanks to some important technological breakthroughs in recent years (Fig. 1). High-dimensional cytometry analyses, such as mass cytometry (Ornatsky et al. 2008) and high-dimensional fluorescence-based cytometry (Bendall et al. 2012) can allow as many as 50 parameters to be measured in individual cells, targeting a variety of cellular targets such as surface proteins, intracellular proteins, mRNA-molecules and secreted proteins (Brodin 2018). There are differences among these modern cytometry methods with flow cytometry leading on speed and their massive throughput as well as their ability to sort viable cells, while mass cytometry allows for about twice as many parameters to be measured on individual cells. Both these powerful methods allow for millions of cells to be analyzed at relatively low cost and consequently researchers are able to capture all the different cell populations present in blood or other tissues. This is a critical point, because such analyses enable inferences of cell–cell relationships during a particular immune response to be unpacked. Plasma protein profiling is another key measurement type in modern immunology targeting cytokine levels, inflammatory mediators and metabolites to be profiled at scale. Currently, hundreds of proteins can be measured with antibody-based assays, either quantified by fluorescence-based approaches or PCR-based approaches (Lundberg et al. 2011). Sequencing-based methods have exploded in popularity and potency in the last decade and now allow not only for entire genomes to be analyzed at scale and reasonable cost, but also mRNA-profiling at single-cell resolution enabling the generation of atlases of cell types across human tissues to be collated (Regev et al. 2017). Moreover, these methodologies now also allow for epigenetic traits such as chromatin accessibility to be analyzed across thousands of individual cells, leading to the understanding of regulatory programs active in such cells (Satpathy et al. 2018). Moreover, the analyses of variable receptors in adaptive lymphocytes using next-generation sequencing allow us to understand immune responses to vaccines and microbial antigens by detecting expanded clones of T and B cells and in some fortunate cases, even leading to the identification of the targeted microbe and antigen (Davis and Boyd 2019). Sequencing-based methods are now also merging with the cytometry-based readouts to generate combined multiomics-profiles of protein expression, mRNA expression and potentially also simultaneous epigenetic traits and immunoreceptor identification, all based on oligo-coupled antibodies, and sequencing-based readouts allowing very complex and comprehensive profiles to be generated for individual immune cells (Mimitou et al. 2019). Although these measurements are very expensive and still mostly for in-depth characterization of small, well-defined immune cell populations, in contrast to upfront immunomonitoring applications, they are indicative of an interesting future development where every individual cell in a sample could be analyzed with respect to its clonality, protein expression, functional state and regulatory pattern. Such datasets will surely help us understand immune system function and regulation in much more details than currently possible. One point of caution is warranted with all these methods that collect high-parameter datasets and this relates to the statistical power required to reliably distinguish biologically relevant associations from spurious patterns. As with any high-dimensional datasets and often small sample sizes from human subjects, correcting for multiple hypotheses testing is critical and carefully assessing the effect sizes of identified associations and maintaining a sound skepticism in general is important to avoid being mislead by spurious results. Another important benefit of studies in humans is the ability for longitudinal monitoring, which can be really helpful in this case.

## Layers of investigation in human immunology

**Fig. 2:**
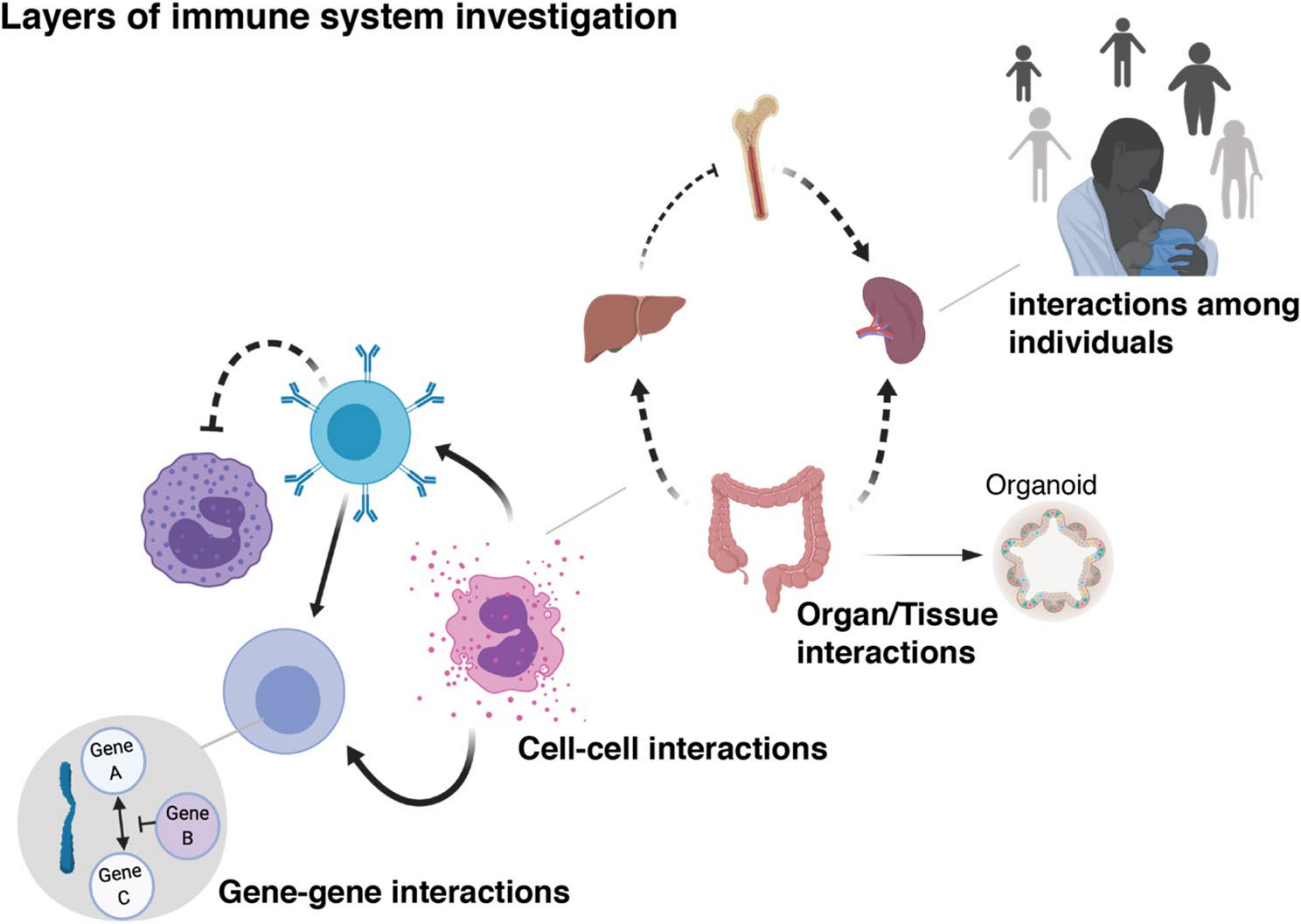
The human immune system can be investigated at multiple levels. Most studies to date have involved gene–gene regulation within populations, but more and more attention is also shifting towards cell–cell interactions within the system. The organismal level of interactions is still limited by technical challenges of longitudinal tissue sampling in humans. Population studies and analyses of inter-personal relationships have long been investigated in the field of epidemiology

The immune system, its specialized cells and the proteins that perform biological functions, can be visualized and studied at different scales. To date, most analyzes in humans have focused on the inner working of immune cells, the genes expressed and how sequence variants in these genes affect cellular function (Fig. 2). There is a lot of interesting interactions among such components to be learned still, and regulatory mechanisms underlying immune system conditions are still under scrutiny. However, with the advent of the advanced technologies described above, offering high-dimensional, and even multiple layers of data from individual cells, we are now beginning to address the next layer of regulatory interaction in the human immune system, the cell–cell interactions (Fig. 2). Understanding how cells stimulate and regulate each other and how this translates into various kinds of immune responses and how such cellular circuits differ among individuals, with different genetic and environmental origins, and over the course of life, is a current frontier in the field of human systems immunology. Each tissue and organ have their own specific niches that are adapted to the organ-specific challenges and needs. As such, the cell–cell interaction patterns are likely to differ across tissues, and a challenge for the future will be to understand how this next layer of regulation—the organismal level, with its interactions between tissues, shape human immune function and determine immune competence at organism level. At the moment, sampling humans across multiple tissues is only possible using cadaver donors, and such studies are beginning to provide interesting insights into the tissue-specific immune cell compartments and niches in such human donors (Meng et al. 2017; Yudanin et al. 2019).

Although these analyses are important to map cell composition and states unique to different tissues, they do not allow for dynamical changes within an individual tissue over time to be studied. Such analyses would require longitudinal samples currently not possible to obtain from most human tissues. Advances in the use of fine-needle aspiration (Austin et al. 2005), and the continued development of more detailed analyses using small sample volumes (Olin et al. 2018) with better sample preparation methods (Brodin et al. 2019), will be required to enable such longitudinal tissue samplings and analyses of organismal immune regulation in humans (Fig. 2).

A proxy approach, allowing dynamical investigation of tissue-specific immune responses, is the use of human organoid cultures, where some aspects of the tissue-specific immune system can be ascertained (Clevers 2016). Some of these model systems can now be used to investigate local immune responses to microbial pathogens, for example, in intestinal and lung organoid cultures (Heo et al. 2018), and tonsil organoids can be used to investigate antigen-specific T- and B-cell responses in vitro (Wagar et al. 2018). Such tools promise to offer more insights into vaccine responses and immune responses to tumors in humans and will allow dynamical investigation into regulatory properties with tissue-specific niches.

A final layer of immune system investigation is that of the inter-individual relationships relevant to immunity, such as the passive antibody transfer from mothers to their offspring during pregnancy and its determinants on infectious disease susceptibility (Pou et al. 2019). The indirect protection of individuals within a population of largely immune individuals, i.e., herd immunity, is another important feature of the inter-individual relationships that determine our disease risk. Although much of our immune system’s composition and functional properties are shaped by evolution under the strong pressure of infectious disease (Quintana-Murci et al. 2007), much of its variation is also explained by environmental factors such as nutrition, infections and our symbiotic microbes (Brodin et al. 2015). Many of these environmental factors are the results of human interaction, leading to increasing similarities among individuals living together (Carr et al. 2016) and individuals with similar chronic viral infections such as Cytomegalovirus (Patin et al. 2018). All in all, the regulation and function of the human immune system can only be understood from analyses at multiple levels of regulation and novel technologies reviewed in this manuscript are enabling more and more such analyses and hopefully in the not too distant future, this will help us interpret, diagnose and modulate more of the many human conditions involving the immune system.
